# Nosocomial outbreak of imipenem-resistant *Pseudomonas aeruginosa *producing VIM-2 metallo-β-lactamase in a kidney transplantation unit

**DOI:** 10.1186/1746-1596-6-106

**Published:** 2011-10-28

**Authors:** S Hammami, I Boutiba-Ben Boubaker, R Ghozzi, M Saidani, S Amine, S Ben Redjeb

**Affiliations:** 1 Laboratoire «Résistance aux Antimicrobiens », Faculté de Médecine de Tunis Université Elmanar, 15 Rue Djebel Akhdhar - La Rabta - 1007 Bab Saâdoun - Tunis, Tunisie; 2 Laboratoire de Microbiologie, Hôpital Charles Nicolle, Boulevard 9 Avril, 1006, Tunis, Tunisie

**Keywords:** pulsed-field gel electrophoresis, carbapenem, *P. aeruginosa*, epidemiology

## Abstract

**Background:**

Twenty four non replicate imipenem resistant *P. aeruginosa *were isolated between January and November 2008, in the kidney transplantation unit of Charles Nicolle Hospital of Tunis (Tunisia). This study was conducted in order to establish epidemiological relationship among them and to identify the enzymatic mechanism involved in imipenem resistance.

**Methods:**

Analysis included antimicrobial susceptibility profile, phenotypic (imipenem-EDTA synergy test) and genotypic detection of metallo-β-lactamase (MBL) (PCR), O-serotyping and pulsed-field gel electrophoresis.

**Results:**

All strains showed a high level of resistance to all antimicrobials tested except to colistin. The presence of MBL showed concordance between phenotypic and genotypic methods. Sixteen isolates were identified as VIM-2 MBL-producers and 13 of them were serotype O4 and belonged to a single pulsotype (A).

**Conclusions:**

This study describes an outbreak of VIM-2-producing *P. aeruginosa *in a kidney transplantation unit. Clinical spread of *bla*_VIM-2 _gene is a matter of great concern for carbapenem resistance in Tunisia.

## Background

*Pseudomonas aeruginosa *is a frequent nosocomial pathogen that causes a wide range of opportunistic infections and nosocomial outbreaks [1-3]. Its high intrinsic resistance to antibiotics and ability to develop multidrug resistance pose serious therapeutic problems. Four broad-spectrum β-lactams, such as carbapenems, are potential drugs for the therapy of infections caused by *P. aeruginosa *[4,5]. However, the increasing use of these compounds has resulted in the emergence of carbapenem-resistant *P. aeruginosa *isolates, limiting treatment options [6,7]. Most carbapenem resistance is due to impermeability, which arises via loss of the OprD (D2) porin, but carbapenem hydrolysing metallo-β-lactamases (MBLs) are increasingly reported [8]. Genes encoding MBLs are located as cassettes in integrons that provide them with the potential for expression and dissemination [9,10]. To date, nine MBL types, namely, IMP-like[9], VIM-like[9], SPM-1[11], GIM-1[12], SIM-1[13], AIM-1[14], KHM-1[15], NDM-1[16] and DIM-1[17], have been identified in Gram negative bacilli. Worldwide, the IMP and VIM types are the most commonly detected MBLs in *P. aeruginosa *[9,10]. VIM-type MBLs are predominant in the Mediterranean region [9,10].

At Charles Nicolle hospital of Tunisia, since November 2002, VIM-2 producing *P. aeruginosa *has been isolated, mainly in surgery and intensive care unit [18]. In 2008, an increasing rate of imipenem resistance in *P. aeruginosa *was observed in the kidney transplantation unit. The aim of the present study was to determine the occurrence of MBL genes among imipenem resistant isolates and to establish epidemiological relationship among them.

## Methods

### Patients, Bacterial strains, Serotyping and susceptibility testing

Twenty four non duplicate imipenem resistant *P. aeruginosa *isolates were recovered between January and November 2008 in the kidney transplantation unit of Charles Nicolle hospital of Tunis. They were isolated from urine (n = 20), cutaneous pus (n = 3) and blood (n = 1).

All samples were taken for microbial diagnosis from 24 different patients [sex ratio 3.8]. The median age of the patients was 51 years [range: 34 to 80 years]. They were admitted in the urology unit (n = 10) and nephrology unit (n = 14).

A retrospective review was conducted for only 15 patients (6 from urology unit, 9 from the nephrology unit). Date of transplantation was mentioned in Table 1. However, no data concerning kind of transplantation and live donator was collected. All patients presented chronic renal failure associated in 13 cases to at least one underlying co-morbidity [High blood pressure (n = 13), systemic lupus erythematosis (n = 1), diabetes (n = 1), chronic glomerulonephritis (n = 5), nephritic syndrome (n = 1) and renal tuberculosis (n = 1)]. In all patients fever was the major symptom. Ceftazidime combination therapy with colistin accounted for all patients. Attributable mortality was 5% of cases.

**Table 1:** Characteristics of imipenem resistant *P. aeruginosa *strains

								MICs							
									
Strains	Date of transplantation	Date of Isolation	Age/Gender	Speciman	EDTA test	PCR VIM-2	Serotype	TIC	TCC	IMP	MEM	CAZ	AZT	FEP	PFGE
1	27/11/2007	04/01/2008	45/F	Cu Pus	-	-	O11	> 2048	> 1024	256	512	2048	> 512	> 256	ND
2	-	23/01/2008	54/M	Urine	+	+	O11	> 2048	> 1024	> 512	> 512	64	> 512	> 256	C
3	NT	06/02/2008	61/M	Urine	-	-	O4	> 2048	> 1024	> 512	> 512	512	> 512	> 256	ND
4	-	10/04/2008	47/M	Urine	+	+	O11	> 2048	> 1024	> 512	> 512	64	> 512	> 256	C
5	7/12/2007	12/05/2008	45/F	Cu Pus	-	-	NA	> 2048	> 1024	> 512	512	1024	> 512	> 256	ND
6	-	20/05/2008	61/M	Urine	+	+	O4	> 2048	> 1024	> 512	> 512	512	> 512	> 256	A
7	-	20/05/2008	52/M	Urine	-	-	O12	> 2048	> 1024	> 512	512	512	> 512	> 256	ND
8	20/12/2007	18/06/2008	45/F	Cu Pus	-	-	O11	> 2048	> 1024	> 512	> 512	512	> 512	> 256	ND
9	-	24/06/2008	75/M	Urine	-	-	O4	> 2048	> 1024	512	> 512	1024	> 512	> 256	ND
10	NT	04/07/2008	63/M	Urine	+	+	O4	> 2048	> 1024	> 512	> 512	1024	> 512	> 256	A
11	08/07/2008	09/07/2008	34/M	Urine	+	+	O4	> 2048	> 1024	> 512	> 512	1024	> 512	> 256	A
12	-	21/07/2008	52/M	Urine	+	+	O4	> 2048	> 1024	> 512	> 512	1024	> 512	> 256	A
13	-	26/07/2008	54/M	Urine	-	-	O4	> 2048	> 1024	> 512	512	1024	> 512	> 256	ND
14	-	02/08/2008	76/M	Urine	+	+	NA	> 2048	> 1024	> 512	512	64	> 512	256	D
15	16/07/2008	02/08/2008	42/M	Urine	+	+	O4	> 2048	> 1024	> 512	> 512	256	> 512	> 256	A
16	10/08/2008	21/08/2008	52/M	Urine	+	+	O4	> 2048	> 1024	> 512	512	512	> 512	> 256	A
17	18/08/2008	26/08/2008	52/M	Urine	+	+	O4	> 2048	> 1024	> 512	> 512	512	> 512	> 256	A
18	-	03/10/2008	20/M	Urine	+	+	O4	> 2048	> 1024	> 512	32	64	> 512	> 256	A
19	28/08/2008	06/10/2008	80/M	Urine	-	-	NA	> 2048	> 1024	> 512	> 512	4	> 512	> 256	ND
20	-	01/11/2008	54/M	Urine	+	+	O4	> 2048	> 1024	> 512	> 512	512	> 512	> 256	A
21	17/10/2008	05/11/2008	31/M	Urine	+	+	O4	> 2048	> 1024	> 512	> 512	512	> 512	> 256	A
22	05/11/2008	17/11/2008	34/F	Blood	+	+	O4	> 2048	> 1024	> 512	> 512	512	> 512	> 256	A
23	15/11/2008	17/11/2008	34/F	Urine	+	+	O4	> 2048	> 1024	> 512	> 512	512	> 512	> 256	A
24	10/09/2008	22/11/2008	52/M	Urine	+	+	O4	> 2048	> 1024	> 512	> 512	1024	> 512	> 256	A

TIC: Ticarcillin; TCC: Ticarcillin-clavulanic acid; IMP: Imipenem; MEM: Meropenem; CAZ: Ceftazidim; AZT: Aztreonam; FEP: Cefepime; -: clinical data Not found; NT: No Tranplantation; Cu Pus: Cutaneous Pus; NA: Non Agglutinable; ND: Not determined.

Bacterial identification was performed using ApiNE (bio-Mérieux, Marcy-l'Etoile, France). O-serotyping was determined by slide agglutination test using polyvalent antisera and 16 monovalent antisera numbered O1-O16 according to the manufacturer's instructions (Bio-Rad, Marnes-La-Coquette, France).

Antimicrobial susceptibility was tested with the agar disk diffusion method according to the CLSI guidelines [19]. The MICs of ticarcillin, ticarcillin + clavulanic acid, ceftazidime, aztreonam, cefepime, imipenem and meropenem were determined using the dilution method in Mueller Hinton agar according to the CLSI guidelines [19]. *E. coli *ATCC 25922 and *P. aeruginosa *ATCC 27853 were used as control strains.

To detect MBL production, the imipenem-EDTA synergy test was used [20]. An enlargement of the inhibition zone of imipenem facing the disc of EDTA was considered as a positive test.

### PCR amplification

For the detection of *bla*_VIM _and *bla*_IMP _genes PCR experiments were performed using consensus primers as described previously [21]. Primers specific for *bla*_VIM-2 _were also used [22].

### Clonal relationship by Pulsed-Field Gel Electrophoresis (PFGE)

Molecular typing of MBL producing isolates was carried out, as described previously by Pulsed-Field Gel Electrophoresis (PFGE) using *Spe*I restriction endonuclease [23]. Clonal relationships based on PFGE patterns were interpreted according to the criteria proposed by Tenover et al [24].

## Results and discussion

Carbapenems are potent agents against multiresistant Gram negative bacilli, including *P. aeruginosa*, but their efficacy is increasingly compromised by the emergence and the worldwide dissemination of carbapenem resistance strains, which are implicated in large outbreaks as described in many countries [1,25]. In Tunisia, frequencies of imipenem resistant *P. aeruginosa *varies between 16% and 37.6% [26-28]. At Charles Nicolle Hospital of Tunis, their frequency was stable until 2004 (1%), but increased dramatically from 2005 (25%). They were mainly isolated in surgery and intensive care unit [18], but in 2008, an increasing rate of multidrug-resistant *P. aeruginosa *was observed in the kidney transplantation unit. All strains exhibited a multidrug-resistant phenotype; they were resistant to antipseudomonal β-lactams (including aztreonam), aminoglycosides and fluoroquinolones. They remained susceptible only to colistin which is used for the treatment of our kidney transplanted patients despite its renal toxicity [29]. MICs results are shown in table 1. All strains showed a high level of resistance to all β-lactams, particularly to carbapenems (> 512 μg/ml). Only 16 strains (67%) were positive according to the imipenem-EDTA synergy test, suggesting the presence of MBLs. The acquisition of a MBL gene alone does not necessarily confer elevated level of resistance to carbapenems. Indeed, secondary changes in regulatory system of MBL gene expression, outer membrane permeability, active efflux systems in bacterial membrane, and/or multiplication of structure gene might well be implicated in acquisition of high-level carbapenem resistance [30,31]. Aztreonam is the only β-lactam that may remain fully active against MBL producers [8], however all our strains were resistant to this β-lactam, suggesting the occurrence of other mechanisms of β-lactam resistance [32]. The eight MBL negative strains were also resistant to all antibiotic tested (Table 1), but the mechanism involved in the resistance has not been further examined.

The 16 MBLs-producer strains were positive for *bla_VIM-2 _*gene and none strain harboured the *bla*_IMP _gene (Table 1). In Tunisia, the most common MBL identified VIM-2 in accordance with the actual situation worldwide [9,33,34]. Historically, the first reports of MBLs genes were VIM-2 and VIM-4 types in *P. aeruginosa *[18,35] and *K. pneumoniae *[36] respectively.

Serotyping identified 3 serotypes: O4 (n = 16), O11 (n = 4) and O12 (n = 1) (table 1). Only 2 strains were nontypeable with monovalent antisera. In Tunisia [37] as well as in many European countries [38], it has been repeatedly demonstrated over the past 20 years that serotypes O11 and O12 dominate among multiresistant *P. aeruginosa *isolates. The 16 MBL-positive strains were divided into 3 pulsotypes designed A (n = 13), B (n = 2) and C (n = 1) (Table 1). The 13 strains of pulsotype A were of serotype O4. These results imply that the dissemination of VIM-2 in our kidney transplantation unit was mainly due to the of spread clonal strains, however, unrelated VIM-2-harboring strains occurred. Outbreaks of VIM β-lactamase-producing *P. aeruginosa *have been also reported in Greece [3], Italy [2] and Kenya [39], but there is still very limited knowledge on the epidemiology of MBLs in Africa.

The emergence of acquired MBLs among *P. aeruginosa *represents an epidemiological risk for at least two reasons: firstly, MBLs confer resistance not only to carbapenems but to virtually all β-lactams and are frequently associated with resistance to aminoglycosides; and secondly, genes encoding for MBL enzymes are most commonly carried on mobile genetic elements (integrons, plasmids, transposons) that can spread horizontally among unrelated strains [9,18].

Indeed, the *bla*_VIM-2 _gene was found in strains of different genotypes (A, B and C), reflecting its ability to transfer from one bacterium to another.

## Conclusion

In conclusion, we found an outbreak of VIM-2-producing *P. aeruginosa *in a kidney transplantation unit. Thus, *bla*_VIM-2 _gene may have been spreading in Gram negative rod in Tunisia. This emphasizes the necessity of early recognition of MBL producing isolates, rigorous infection control, and restricted clinical use of broad-spectrum β-lactams including carbapenems.

## List of abbreviations

MBL: metallo-beta-lactamase; PFGE: Pulsed-Field Gel Electrophoresis.

## Competing interests

The authors declare that they have no competing interests.

## Authors' contributions

SH designed the study and wrote the manuscript. IBBB, RG, MS, SA and SBR performed critical reading of manuscript and supervision. All authors have read and approved the final manuscript.
